# Bridging the gaps: building a labor force to meet long-term care needs

**DOI:** 10.1093/haschl/qxaf217

**Published:** 2025-11-21

**Authors:** Andrea Odinakachukwu Orji

**Affiliations:** University of Michigan Medical School, 1301 Catherine St., Ann Arbor, MI 48109, USA; Gerald R. Ford School of Public Policy, Joan & Sanford, Weill Hall, 735 S State St, Ann Arbor, MI 48109, USA

**Keywords:** long-term care, direct care workforce, Medicaid

## Abstract

The long-term services and supports (LTSS) system is intended to grant Americans, requiring care that extends beyond what can be provided in a hospital and/or primary care settings alone, access to the appropriate caretakers and facilities. Federal policy has struggled to ensure sustainable access to LTSS and the workforce that provides it. In particular, Medicaid beneficiaries are required to have access to these services but still face difficulties in obtaining them. Long term care provision has traditionally been institutionally based due to funding mandated through Medicaid, but the formalized workforce cannot keep pace with an aging population. Many rely instead on informal caregivers (ie, family, friends, etc.) for their care. While populations requiring long term care prefer to receive care from the comfort of their own communities, informal caregivers are rarely compensated and often forced to juggle employment and caregiving duties. This commentary explores how mandated funding for home and community-based services (HCBS) may improve access to LTSS by mobilizing an existing informal workforce.

America's elderly population is growing rapidly. Baby Boomers life expectancy is 78, with the whole generation estimated to surpass age 65 by 2030; meanwhile, the 85 and older population is expected to triple by 2050.^1^ Decades of research have generated advances in medicine, technology and public health that have lengthened average lifespans compared to when Medicaid and Medicare were originally introduced.^2^ However, while attempts to prolong life continue to garner funding with ease, long term care (LTC) and the direct care workforce (DCW) fueling it does not. As LTC needs grow alongside an aging population, it is crucial that investments in this workforce can ensure appropriate capacity to provide care that keeps pace with population needs.

Several healthcare sectors suffered serious workforce losses following COVID-19, but the caregiving industry took a particularly hefty hit.^3^ Overall health sector employment surpassed pre-pandemic levels in all except two sub-sectors by 2024: elderly care (1% decrease) and skilled nursing facilities (8.3% decrease).^4^ Underemployment in these sectors is worsened by many states’ Certificate of Need laws which effectively restrict the growth of the institutional care sector and its corresponding workforce to mitigate unnecessary spending despite growing long-term care needs.^5^

Family caregivers, working within their dependents’ homes and communities, provided an alternative. They quickly became an unpaid means of addressing gaps exposed by the pandemic, and provided value that was temporarily recognized through the provision of cash payments, tax credits and increased federal match rates for Medicaid in the American Rescue Plan, a financial relief bill meant to revitalize the post COVID economy.^6^ However, recognition of the cost effective work provided through Home and Community Based Services (HCBS) was fleeting.^5^ As block grants from the COVID era expired, discussions regarding funding renewal were scarce despite the success demonstrated by HCBS. As such, institutional LTSS continues to be the only LTC provision mandated through Medicaid.

The reality of the current DCW is two-fold:

1. Availability of informal/family caregivers is waning as the overall U.S. population continues to age rapidly,^3^ and
2. Our formal caregiver workforce is inadequate to fill growing LTSS needs as the informal sector declines.

Medicare currently covers 100 benefit days of skilled nursing per billing period and 28 hours of weekly home care services.^7^ Informal caregivers often bear remaining care needs. Medicaid continues to be the primary payer for LTSS, followed closely by personal expenditures, but fails to allocate mandatory funding for HCBS despite ongoing interest from beneficiaries. Home based LTSS is offered primarily through 1915 waivers, which require beneficiaries to check additional boxes and re-assert compliance with everchanging eligibility criteria. Furthermore, waiver-based access is plagued by endless waitlists as HCBS requests outpace the care that available waiver funds can offer.

It is long overdue for HCBS to be a mandated Medicaid service. Doing so would require a portion of federal Medicaid funding received by states to be allocated to HCBS care compensation. This would eliminate the need for the current waiver system, which limits benefit coverage and waitlist access across state lines depending on concordance of eligibility criteria.^7^ While access to long-term care would be expanded, the lingering question of quality assurance remains. Quality measure sets are one aspect of the waiver system that could be retained and modified.^8^ They have traditionally been used to assess home-based care, including that provided by informal caregivers. Though admittedly imperfect in assessment scope, these measure sets could provide a good baseline to ensure tracking of quality and efficiency metrics in a changing system.

It's important to recognize that mandating HCBS as a space for bridging gaps in the DCW is just one step toward building a more sustainable long-term care system. While the informal caregiving workforce dwindles, it is critical that we also use this bridging space and time-period to build a labor sector that the formal direct care workforce will want to engage.

Moving forward, solutions must incentivize the growth of informal and formal caregiving workforces and HCBS is precisely where a transition between the two can occur somewhat smoothly. Mandating this care would allow informal caregivers to have consistent access to funding to replace the estimated $600 billion of unpaid labor they contribute annually.^9^ Additionally, HCBS cost savings were an estimated $26 billion from avoidable hospitalizations alone in 2017, and could be used to provide formal caregivers with benefits and professional development opportunities that they have not traditionally received.^6^

Simultaneous reinforcement of these workforces is being harmed by current legislation. Though the Big Beautiful Bill increases the number of 1915 waivers granted to each state for HCBS, it significantly diminishes funding for Medicaid (particularly in states with a history of Medicaid expansion) and delays processes intended to ensure adequate staffing and reimbursement of direct care workers in institutional facilities.^10^ Failure to move beyond a one-pronged solution will limit our ability to craft a long-term care system that adequately serves the U.S. population.

America's DCW is projected to increase to 6 million persons by 2032, but this number falls short of estimated needs.^6^ However, proper investment in HCBS has the potential to fill current gaps and fuel growth in the field at large. Ideally, by the time the family caregiver population has been outpaced by the number of people requiring care, the formal caregiver workforce will have built the capacity to meet remaining needs.

Many facets of the current LTSS system need serious reform. If properly attended to, the caregiving workforce may prove a strong foundation for a more sustainable system. This is particularly important as current attacks on Medicaid further destabilize the existing system. A robust and growing workforce means an increased likelihood of dependents receiving the necessary care at a lower cost. While innovations that increase access to long-term care should be given high priority, without a workforce to provide affordable care, relevant stakeholders won’t have a system to invest in. Investing in HCBS as a means of smoothing an unavoidable labor force transition creates potential for supporting accessibility on both fronts and is an opportunity that should not be ignored.

## Supplementary Material

qxaf217_Supplementary_Data
